# Kidney Transplantation over 65 Years: Clinical and Immunological Long-Term Outcomes—Single Center Experience

**DOI:** 10.3390/geriatrics11030066

**Published:** 2026-06-02

**Authors:** Lucia Federica Stefanelli, Marianna Alessi, Martina Cacciapuoti, Rime Khalf, Dorella Del Prete, Francesca Katiana Martino, Lorenzo A. Calò, Federico Nalesso

**Affiliations:** Nephrology, Dialysis and Transplantation Unit, Department of Medicine, University of Padua, 35128 Padua, Italy; luciafederica.stefanelli@unipd.it (L.F.S.); marianna.alessi@aopd.veneto.it (M.A.); martina.cacciapuoti@phd.unipd.it (M.C.); khalfrime@gmail.com (R.K.); dorella.delprete@unipd.it (D.D.P.); francescakatiana.martino@unipd.it (F.K.M.); federico.nalesso@unipd.it (F.N.)

**Keywords:** kidney transplant, older kidney transplant recipients, kidney transplant complications in older recipients, clinical outcomes, immunological outcomes

## Abstract

**Background/Objectives**: Kidney transplantation (KT) in older recipients remains challenging due to age-related conditions such as frailty and comorbidities, as well as immunological changes related to immunosenescence, which expose older KTRs to a higher risk of infection and infection-related complications. The aim of this study was to evaluate clinical and immunological outcomes in older KTRs, analyzing the incidence of cardiovascular, infective, and neoplastic complications, as well as graft and patient survival and the associated risk factors. **Methods**: This monocentric study includes 157 KTRs aged over 65 years, followed at the Transplant Ambulatory of Padua University Hospital and transplanted between January 2013 and December 2023. Clinical and immunological outcomes were evaluated, including surgical complications, incidence of delayed graft function (DGF), and renal function at 1, 3, and 5 years after KT. **Results**: Patient survival rates were 96%, 91.5%, and 71.6% at 1, 3, and 5 years after KT, respectively, while graft survival rates were 94%, 87%, and 68%. Major complications were malignancies (40.1%), cardiovascular disease (33.1%), and bacterial infections (22%). In the multivariate analysis, donor age and history of malignancy were identified as independent risk factors for mortality (*p* = 0.048 and *p* = 0.056, respectively). Kaplan–Meier survival analysis confirmed donor age as the only significant risk factor for patient survival. Regarding graft survival, multivariate analysis identified hypertension as an independent risk factor for graft failure (*p* = 0.009), while Kaplan–Meier analysis showed diabetes (*p* = 0.040) and single-kidney transplantation (*p* = 0.003) as significant risk factors. **Conclusions**: KT in older recipients represents a safe and beneficial therapeutic option, offering favorable patient and graft survival outcomes. However, this epidemiological study highlights the need for personalized follow-up strategies and improved prognostic assessment in older KTRs.

## 1. Introduction

Kidney transplantation (KT) is certainly the most effective substitutive treatment for end-stage kidney disease (ESKD), prolonging patients’ survival and improving quality of life (QOL) compared to dialysis [1]. Moreover, the number of patients developing kidney failure has increased in recent years due to population aging, as well as the demand for KT among older individuals [2]. In Europe, nearly 17–20% of kidney transplant recipients (KTRs) are over 70 years of age, demonstrating that age is no longer considered an absolute contraindication to kidney transplantation [3].

Although survival benefits and improved QOL may also apply to patients over 60 years of age, regardless of donor characteristics, aging may be associated with multiple comorbidities, frailty, and cognitive impairment, which may represent significant barriers to KT and negatively affect outcomes and patient status [4]. In addition, the older population commonly presents immune dysfunction/deficiency, and these conditions may expose elderly patients to a higher risk of complications, including infections and post-transplant malignancies [5]. For older patients considered for KT, the risks and benefits of transplantation should therefore be carefully compared with those of dialysis [6].

The decision to include an older patient in the transplant waiting list should therefore prioritize individual patient characteristics and expected outcomes.

The aim of this retrospective study was to evaluate clinical and immunological outcomes in older KTRs, analyzing the incidence of cardiovascular, infectious, and neoplastic complications, as well as graft and patient survival, and the independent risk factors associated with these outcomes. In addition, with this study, we further investigated this topic to assess whether KT is a safe option for the older population and to identify the most relevant factors to consider when approaching older patients in order to personalize clinical decision-making.

## 2. Materials and Methods

### 2.1. Study Design

This was a single-center, longitudinal, retrospective study including kidney transplant recipients aged >65 years who underwent transplantation at the Padua Kidney–Pancreas Transplant Center, University Hospital of Padua (Padua, Italy), between 1 January 2013, and 31 December 2023.

A total of 157 patients who underwent either single or dual kidney transplantation were included. Outcomes from both living and deceased donors were evaluated.

Exclusion criteria included patients followed at other centers after kidney transplantation due to incomplete outcome data.

### 2.2. Patients

Demographic, clinical, and immunological data were collected from kidney transplant recipients up to 31 December 2023.

All data were extracted from medical records and included patients’ sociodemographic characteristics, transplant history, dialysis duration before transplantation, and renal allograft conditions, including rejection episodes and comorbidities, causes of ESKD, type of renal replacement therapy, immunosuppressive regimen, and immunological profile.

Outcome data included graft and patient survival at 1, 3, and 5 years after transplantation.

Patients underwent regular follow-up for immune status and disease progression monitoring.

Ethical review and approval were waived for this study, as permitted for the retrospective nature of this study. However, patients were not exposed to any risk by the irreversible anonymization of data, which prevented any possible disclosure of sensitive data, ensuring subjects’ privacy.

### 2.3. Outcomes Definition

Elderly candidates undergo the same evaluation process applied to younger recipients. Candidate selection is mainly based on overall clinical status and comorbidity burden rather than chronological age alone. Severe cardiovascular disease and advanced peripheral vasculopathy represent major exclusion criteria for kidney transplantation in elderly candidates.

Older patients were defined as those aged ≥65 years, according to the United Nations definition [7]. Outcomes included recipient and graft survival at 1, 3, and 5 years after transplantation. Additional outcomes included acute rejection episodes during follow-up, defined according to the Banff classification, delayed graft function (DGF), defined as the need for at least one hemodialysis session during the first week after transplantation, and death-censored graft failure, defined as return to dialysis or transplantation.

We also evaluated new-onset malignancies, post-transplant infections, and metabolic complications during follow-up. Cardiovascular complications were defined as the occurrence of at least one major adverse cardiovascular event (MACE).

Infectious complications included bacterial, viral, or fungal infections, both early and late infections.

Cardiac events included myocardial infarction, ischemic heart disease, new-onset congestive heart failure, and arrhythmias.

For CV and malignancy screening, due to the known increased risk of skin cancer in KTRs, dermatological surveillance every 6 months is performed. For other malignancies, the same screening protocols recommended for the general population are adopted, including chest X-ray every 2 years, annual abdomen ultrasound, fecal occult blood test (FOBT), PAP Test and mammography according to current guidelines. For CV screening, we recommend annual cardiological evaluation including echocardiography according to our local protocol.

Surgical complications, defined as postoperative events directly related to the surgical procedure requiring intervention, were categorized into three groups: vascular (including renal graft vessel thrombosis, renal artery stenosis, iliac artery dissection, iliac artery thrombosis, and pseudoaneurysm), urological (urinary leakage, ureteral obstruction, lymphocele), and wound-related complications (wound dehiscence, infection, and delayed healing).

Renal function was assessed using serum creatinine levels expressed in µmol/L.

### 2.4. Immunosuppression

Induction immunosuppressive therapy was administered to all patients. It consisted of either two doses of anti-IL-2 receptor monoclonal antibody (basiliximab, 20 mg on day 0 and day +4 after transplantation) or rabbit anti-thymocyte globulin (ATG, 2.5 mg/kg/day) for 5 consecutive days in immunologically high-risk recipients (defined as Panel Reactive Antibody (cPRA) >25% and/or ≥1) previous kidney transplant lost due to immunological causes), as well as in recipients of kidneys from donation after circulatory death (DCD).

Maintenance immunosuppression consisted of triple therapy with tacrolimus or cyclosporine (calcineurin inhibitors, CNIs), mycophenolate or mTOR inhibitors, and low-dose steroids.

Target trough levels were 5–8 ng/mL for tacrolimus, 50–80 ng/mL for cyclosporine, and 3–4 ng/mL for everolimus.

### 2.5. Statistical Analysis

Statistical analyses were performed using SPSS software version 29 (IBM Corp., Armonk, NY, USA).

Categorical variables were expressed as absolute numbers and percentages, while continuous variables were reported as mean ± standard deviation (SD) or median and interquartile range (IQR), according to their distribution.

Kaplan–Meier survival analysis was used to estimate patient and graft survival, and differences between groups were compared using the log-rank test.

Binomial logistic regression was used to calculate odds ratios (ORs), and Cox proportional hazards regression analysis was performed to estimate hazard ratios (HRs) for patient and graft outcomes.

A *p*-value < 0.05 was considered statistically significant.

## 3. Results

### 3.1. Demographics

A total of 157 patients were included in the study. Among them, 105 (66.9%) were male, and 55 (35.0%) underwent dual kidney transplantation.

The mean age at transplantation was 69.0 ± 3.25 years (range 67–72), and 72 patients (45.9%) were ≥70 years old.

In this cohort, 33 patients (21.0%) had ESKD due to glomerulonephritis, and in 31 patients (19.7%), the cause was unknown due to a late referral.

The median dialysis vintage was 20 months (IQR 9–36).

From an immunological perspective, 10 patients (6.4%) had PRA > 25%.

Induction therapy was most commonly performed with ATG (137 patients, 87.3%), while the most frequent maintenance therapy in combination with CNIs included mTOR inhibitors (98 patients, 62.4%).

The median donor age was 76.0 years (IQR 69.5–81), and 48 donors (30.6%) were male. Baseline characteristics are summarized in Table 1.

### 3.2. Clinical Outcomes

Among the 157 recipients, 13 patients (8.3%) developed post-transplant hypertension, and 31 (19.7%) developed diabetes mellitus (NODAT).

Cardiovascular complications occurred in 52 patients (33.1%), while 63 patients (40.1%) developed post-transplant malignancies.

Bacterial infections were the most frequent infectious complication, affecting 34 patients (22.0%).

Delayed graft function (DGF) occurred in 17 patients (10.8%), and acute rejection was observed in six patients (3.8%) during follow-up.

Surgical complications were reported in 58 patients (36.6%). The complications evaluated included mainly vascular (including renal graft vessel thrombosis, renal artery stenosis, iliac artery dissection, iliac artery thrombosis, and pseudoaneurysm) and urological (urinary leakage, ureteral obstruction, lymphocele) complications.

The main outcomes are summarized in Table 2.

The median eGFR level at 1 year was 55.4 mL/min/1.73 m^2^ (IQR 70.3–42.9), decreasing to 50.4 mL/min/1.73 m^2^ (IQR 69.8–36.0) at 5 years. In particular, the median eGFR level at 1 year was 58.2 mL/min/1.73 m2 (IQR 77.7–43.2) in dual kidney recipients vs. 54.8 mL/min/1.73 m2 (IQR 67–41.9) in single kidney recipients.

Patient and graft survival, along with creatinine trends over time, are shown in Table 3.

### 3.3. Mortality and Graft Loss

During the follow-up period, 58 patients died (36.9%). Patient survival was 96% at 1 year, 91.5% at 3 years, and 71.7% at 5 years.

In the univariate analysis, cardiovascular complications (*p* = 0.011; OR 2.5), malignancies (*p* = 0.046; OR 2.02), and donor age (*p* = 0.036; OR 1.08) were associated with mortality.

In the multivariate analysis, malignancies showed a borderline association with mortality (OR 3.306, *p* = 0.056), while donor age remained independently associated with mortality (OR 1.158, *p* = 0.008).

Kaplan–Meier analysis showed that PRA > 25% was associated with reduced survival (log-rank *p* < 0.001).

In our cohort, 12 patients (7.6%) developed graft loss.

In the univariate analysis, dialysis vintage (*p* = 0.017; OR 1.022), hypertension (*p* = 0.032; OR 5.083), and acute rejection (*p* = 0.049; OR 6.200) were associated with graft loss.

In the multivariate analysis, only hypertension remained independently associated with graft loss (*p* = 0.009).

Kaplan–Meier analysis identified diabetes mellitus (log-rank *p* = 0.040) and donor type (*p* = 0.003) as significant factors associated with graft survival.

**Figure 1:**
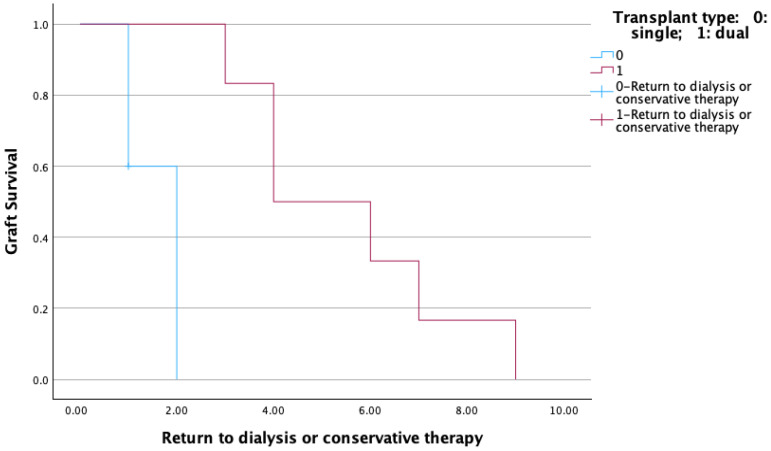
Kaplan–Meier curve for graft survival in relation to transplant type. 0: Single kidney transplant. 1: Dual kidney transplant.

Univariate and multivariate analyses are reported in Table 4, and Kaplan–Meier curves are shown in Figure 1.

## 4. Discussion

Our single-center retrospective study evaluated clinical and immunological outcomes in elderly kidney transplant recipients. We observed favorable short- and mid-term patient survival, with rates exceeding 90% at 3 years, supporting the feasibility of kidney transplantation in this population.

Importantly, donor age emerged as an independent predictor of mortality, highlighting the critical role of donor selection in elderly recipients. In addition, malignancies showed a relevant impact on outcomes, although with borderline statistical significance.

Our findings are consistent with previous studies reporting comparable survival outcomes in elderly recipients. For instance, So et al. [8] reported 5-year patient and death-censored graft survival rates exceeding 75%, while data from the Catalan Renal Registry [9] showed long-term survival around 70% in older KTRs. Similarly, a large meta-analysis from the EAU-YAU group [10] including more than 293,000 transplant recipients confirmed that advanced age alone should not preclude transplantation.

In many KT programs, transplantation teams attempt to match by allocating ECD (expanded criteria donors) organs to older recipients [11]. Compared with organs from standard criteria donors, ECD kidneys are associated with worse graft survival and mortality outcomes, but with increased life expectancy over dialysis in older recipients.

In our multivariate analysis, donor age remained an independent risk factor for mortality. This finding reinforces the importance of donor-recipient matching strategies in elderly patients, where the use of expanded criteria donors (ECDs) must be carefully balanced against the potential impact on long-term outcomes. Furthermore, our study highlights how dual KTs are associated with a marginal benefit in terms of graft survival compared to single-KT deceased donors, as shown by our data (*p* = 0.003). This finding may also be partially explained by the surgical technique adopted for dual kidney transplantation, where both kidneys are implanted ipsilaterally, one cranially and the other caudally in the iliac fossa, a strategy previously associated with reduced operative time and postoperative complications [12].

The high incidence of cardiovascular complications (33.1%) and malignancies, especially non-melanoma skin cancers (40.1%), reflects the clinical complexity of elderly recipients. These findings are in line with previous reports identifying infections and cancers as leading causes of death in this population [13]. This supports the need for tailored immunosuppressive strategies, potentially favoring lower-intensity regimens in frail patients, although further studies are required to balance the risk of rejection.

Although a lack of reported data about postoperative complications has been identified, particularly from a surgical point of view, it is known that older recipients are usually at a greater risk of perioperative complications, including death, mainly due to infection and cardiovascular disease [14].

Some studies have reported that the rate of surgical site infections reached 18.8% and that of urinary fistula 10.1% in patients over 70 years of age [15]. In our cohort, postoperative surgical complications occurred in 36.6% of patients, a higher rate compared to previous reports. However, most complications did not significantly impact graft survival, and the relatively low DGF rate (10.8%) suggests that kidney transplantation remains a feasible option in carefully selected elderly patients.

Regarding renal function in the long-term outcomes, one-year median eGFR values of 55.4 mL/min/1.73 m^2^ and 5-year values of 50.4 mL/min/1.73 m^2^, confirm the stability of renal function during follow-up, in line with that described by Rao et al. [9], who highlighted a five-year graft survival rate of 64% in transplants from donors over 8 years of follow-up time, independently from the leading cause of ESKD, nephropathy.

These results suggest that, even in the presence of a physiological reduction in renal reserve function related to the donor’s age, KT outcomes remain a satisfactory option over time if it is performed under suitable clinical conditions.

A strength point of this study is the large and complete cohort, which included a very large number of transplanted patients during a long-term study period over 10 years of observation, with few missing values and complete follow-up data points, highlighting the potential need of geriatric or frailty assessment and underlying the importance of reporting surgical, clinical and immunological complications as an important part of KT outcomes, particularly in the old-age setting, where it becomes even more relevant when selecting potential candidates.

This study has some limitations. This is a retrospective and single-center study in which a cohort of older recipients who have been considered suitable for KT has not been compared with a younger group. This could have induced a selection bias since the recipients included in the waiting list were probably patients with only a few comorbidities and in good condition to be transplanted, and the donors were selected according to the recipients’ risk to minimize the consequences of a non-functioning kidney graft. Furthermore, frailty is a risk factor for poor post-transplantation outcomes, and markers of frailty are not routinely collected. Other information, such as the severity of disease, is not routinely collected, and prior treatment or interventions of vascular comorbidities are also not reported. Similarly, details regarding the stage and histological types of prior cancers and about the type of donors were also missing. Finally, the timing of the onset of complications was not analyzed, which could have provided additional information on the risk dynamics during long-term follow-up.

However, despite these limitations, we believe that our study may provide further evidence on the impact of the recipient age in KT, and based on the data of our study, it might be suggested that recipient age by itself should not be a criterion to contraindicate KT. In addition, our study provides evidence on the feasibility and outcomes of KT in elderly patients and supports the need for specific predictive models and their evaluation for an appropriate selection of patients.

## 5. Conclusions

Kidney transplantation in elderly patients represents a safe and effective therapeutic option when patients are carefully selected. Despite the higher incidence of complications, patient and graft survival remain satisfactory. These findings support a personalized approach to transplantation in older recipients, focusing not only on survival but also on functional status and quality of life.

## Figures and Tables

**Table 1:** Clinical characteristics of the elderly KTR patients.

Variable	Total
Age at transplantation (years)	69.0 ± 3.25 (67–72)
Age ≥ 70 years, n (%)	72 (45.9%)
Male sex, n (%)	105 (66.9%)
BMI (kg/m^2^)	24.8 ± 2.98 (22.8–27.2)
Pre-transplant dialysis duration (months)	20 (9–36)
PRA > 25%	10 (6.4%)
Induction therapy with ATG, n (%)	137 (87.3%)
Maintenance therapy with mTOR-i, n (%)	98 (62.4%)
Type of donor	
– Single deceased donor	91 (57.9%)
– Living donor	10 (6.4%)
– Dual transplant from elderly donor	55 (35.0%)
Donor age (years)	76.0 ± 9.12 (69.5–81)
Male donor, n (%)	48 (30.6%)
Underlying kidney disease	
– Type 2 diabetes mellitus	16 (10.3%)
– Angiosclerosis	25 (16.1%)
– Glomerulonephritis	33 (21.3%)
– ADPKD	28 (18.1%)
– Obstructive uropathy or reflux	12 (7.7%)
– NDD/other causes	43 (26.5%)

**Table 2:** Main outcomes in the cohort of elderly KTRs.

Outcomes	Total
Mortality during follow-up	58 (36.9%)
Cardiovascular complications	52 (33.1%)
Post-transplant malignancies	63 (40.1%)
Bacterial infections	34 (22.0%)
NODAT	31 (19.7%)
Post-transplant hypertension	13 (8.3%)
Acute rejection	6 (3.8%)
DGF (delayed graft function)	17 (10.8%)
Surgical complications	58 (36.6%)
Length of hospital stay (days)	13 ± 6.39 (11–17.5)

**Table 3:** Patient and graft survival during the follow-up period.

Time (Years)	Graft Survival (%)	Patient Survival (%)	eGFR (mL/min/1.73 m^2^) (IQR)
1	96%	94%	55.4 (70.3–42.9)
3	91.5%	87%	52.3 (66.3–40)
5	71.7%	68%	50.4 (69.8–36)
10	55%	52%	46.9 (62.7–34.7)

**Table 4:** Univariate and multivariate analyses for graft and patient survival.

	Univariate Analysis				Multivariate Analysis			
	Mortality		Graft Loss		Mortality			
	OR	*p*-Value	Exp (β)	*p*-Value	OR	*p*-Value	OR	*p*-Value
Sex	1.042	0.910	0.727	0.642				
Recipient age	0.956	0.984	0.409	0.230				
Donor sex	1.543	0.389	0.727	0.642				
Donor age	1.083	0.036	0.945	0.092	1.158	0.008		
Type of Tx	1.036	0.844	1.385	0.301				
BMI	0.889	0.065	0.903	0.387				
Dialysis type	0.820	0.689	0.517	0.539				
Dialysis vintage	1.007	0.267	1.022	0.017			1.018	0.118
PRA > 25%	1.955	0.367	1.083	0.943				
Transplant number	0.586	0.945	3.125	0.328				
DGF	0.504	0.254	1.659	0.541				
Surgical complications	0.830	0.603	0.353	0.191				
Cardiovascular complications	2.503	0.011	0.674	0.569	0.698	0.644		
Infectious complications	1.154	0.663	0.892	0.835				
Post-transplant hypertension	1.310	0.509	5.08	0.032			17.097	0.009
NODAT	1.316	0.509	0.792	0.772				
Malignancies	2.007	0.046	1.038	0.952	3.306	0.056		
Acute rejection	3.358	0.170	6.200	0.049			3.572	0.312

## Data Availability

The original contributions presented in this study are included in the article. Further inquiries can be directed to the corresponding author.

## References

[1] Ryu J.H., Koo T.Y., Ro H., Cho J.H., Kim M.G., Huh K.H., Park J.B., Lee S., Han S., Kim J. (2021). Better health-related quality of life in kidney transplant patients compared to chronic kidney disease patients with similar renal function. PLoS ONE.

[2] Park B.H., Kil S.Y., Kim Y.N., Shin H.S., Jung Y., Rim H. (2024). Kidney transplantation in the elderly. Korean J. Intern. Med..

[3] McAdams-DeMarco M.A., James N., Salter M.L., Walston J., Segev D.L. (2014). Trends in Kidney Transplant Outcomes in Older Adults. J. Am. Geriatr. Soc..

[4] Thind A.K., Willicombe M., Dor F.J.M.F., Johansson L., Thomas N., Rule A., Goodall D., Levy S., Brice S., Ospalla D. (2025). Frailty Impact on Kidney Transplantation in Older People. Kidney Int. Rep..

[5] Jallah B.P., Kuypers D.R.J. (2024). Impact of Immunosenescence in Older Kidney Transplant Recipients: Associated Clinical Outcomes and Possible Risk Stratification for Immunosuppression Reduction. Drugs Aging.

[6] Schoot T.S., Goto N.A., van Marum R.J., Hilbrands L.B., Kerckhoffs A.P.M. (2022). Dialysis or kidney transplantation in older adults? A systematic review summarizing functional, psychological, and quality of life-related outcomes after start of kidney replacement therapy. Int. Urol. Nephrol..

[7] Chadban S.J., Ahn C., Axelrod D.A., Foster B.J., Kasiske B.L., Kher V., Kumar D.M., Oberbauer R., Pascual J., Pilmore H.L. (2020). KDIGO Clinical Practice Guideline on the Evaluation and Management of Candidates for Kidney Transplantation. Transplantation.

[8] So S., Au E.H.K., Lim W.H., Lee V.W.S., Wong G. (2020). Factors Influencing Long-Term Patient and Allograft Outcomes in Elderly Kidney Transplant Recipients. Kidney Int. Rep..

[9] Rao P.S., Merion R.M., Ashby V.B., Port F.K., Wolfe R.A., Kayler L.K. (2007). Renal transplantation in elderly patients older than 70 years of age: Results from the Scientific Registry of Transplant Recipients. Transplantation.

[10] Artiles A., Domínguez A., Subiela J.D., Boissier R., Campi R., Prudhomme T., Pecoraro A., Breda A., Burgos F.J., Territo A. (2023). Kidney Transplant Outcomes in Elderly Population: A Systematic Review and Meta-analysis. Eur. Urol. Open Sci..

[11] Tsarpali V., Midtvedt K., Lønning K., Bernklev T., Åsberg A., Fawad H., von der Lippe N., Reisæter A.V., Røysland K., Heldal K. (2022). A Comorbidity Index and Pretransplant Physical Status Predict Survival in Older Kidney Transplant Recipients: A National Prospective Study. Transplant. Direct.

[12] Cocco A., Shahrestani S., Cocco N., Hameed A., Yuen L., Ryan B., Hawthorne W., Lam V., Pleass H. (2017). Dual kidney transplant techniques: A systematic review. Clin. Transplant..

[13] Chu N.M., Chen X., Norman S.P., Fitzpatrick J., Sozio S.M., Jaar B.G., Frey A., Estrella M.M., Xue Q.-L., Parekh R.S. (2020). Frailty Prevalence in Younger End-Stage Kidney Disease Patients Undergoing Dialysis and Transplantation. Am. J. Nephrol..

[14] Barbachowska A., Gozdowska J., Durlik M. (2024). Kidney Transplantation in Older Recipients Regarding Surgical and Clinical Complications, Outcomes, and Survival: A Literature Review. Geriatrics.

[15] Cuadrado-Payán E., Montagud-Marrahi E., Casals-Urquiza J., del Risco-Zevallos J., Rodríguez-Espinosa D., Cacho J., Arana C., Cucchiari D., Ventura-Aguiar P., Revuelta I. (2022). Outcomes in older kidney recipients from older donors: A propensity score analysis. Front. Nephrol..

